# Panaritium

**Published:** 1884-03

**Authors:** Theodor Billroth

**Affiliations:** Professor of Surgery in the University of Vienna


### PANARITIUM.

BY THEODOR BILLROTH.

*Professor of Surgery in the University of Vienna.*

[Medical Press and Circular.]

Panaritium is the unfortunate designation of an acute inflammation of the finger that may attack as well the first as the second or third phalanx, and that has its seat in the subcutaneous cellular tissue, whence the name *panaritium subcutaneum*, or in the sheath of the tendons (*panaritium tendinosum*), or, lastly, in the bone (*panaritium ostale*). The disease may arise in consequence of crushing, or wounds, or most frequently from infection; it usually begins with pain, from the degree of severity of which a conclusion may be drawn as to the seat of the inflammation, for in those inflammations that have their origin in the bone, the pains are so intense that the sufferers, as a rule, are disturbed in their rest. The swelling in the case of *panaritium ostale* is so light that it bears no proportion to the agony endured. Such intense pain is produced by the retention of the pus beneath the periosteum. When it raises this up and escapes into the cellular tissues, the pain gradually diminishes. From the commencement of the affection until spontaneous rupture externally takes place, usually from twelve to fourteen days elapse; early incision is therefore a great relief to the patient. Incisions can be made even when the pain is most intense, the patient being narcosed, but the incision must, of course, reach down to the bone. The ostitis of the third phalanx generally ends in either total or partial necrosis of the bone. Three or four weeks elapse before the bone is separated from its surroundings — from the tendons, the articular ligaments, or, in the case of partial necrosis, from the still living bone. During this period new bone is already developed by the raised up periosteum, and even if the sequestrum capsule is not completely tubular, in the course of time the development of the new bone may still be considerable. If an incision has been made, examination with the probe must be resorted to from time to time—about once a week—to ascertain how much of the surface of the bone is free, and if found to be fairly freed from its surroundings, it should be seized with a strong pair of forceps and quickly removed. The wound will now close, but at the same time the formation of new bone will be limited, and in consequence of contraction, to a certain extent only a rudimentary phalanx may be developed

The finger will, of course, be always somewhat deformed; it generally becomes slightly clubbed. The incision is usually made by the side of a tendon; the tendon may already be necrosed, but it is not always so, and consequently it would not be proper to aid the occurrence of necrosis by incision. Spontaneous bursting most frequently takes place around the tip of the finger; there are cases, however, in which it takes place under the nail, or at the root of the nail. In such cases the pain is always intense as the nail is only raised up gradually, and the process of elevation is extremely painful. In cases in which the matrix is affected in the process of perforation, the nail becomes necrosed. As far as the nail looks red, it has life in it, just like hair that remains in the cutis; when matter is collected under the nail it no longer looks red and translucent, but becomes dull, milky colored, and later on it becomes greyish black, a sign that the nail is already dead. Death of the matrix, however, is not always associated with death of the nail; this may occur, but the death of the nail-bed is not a necessary result of the process; the nail-bed may remain, and the nail may be formed anew. One thing remains: the inflamed nail-bed never retains so smooth a form as before; it becomes uneven, knotty, and in consequence the nail is never developed with such beautiful sm othness; it remains uneven, and of a dull, milky color. In the course of years this condition may improve a little, but it never becomes good. Various attempts have been made to restrict this uneven growth of the nail by even compression, or by scraping, but the results are only transient, as the cause of the irregular growth is not removed; the matrix still remains uneven and lumpy. When the pus is originally formed in the subcutaneous cellular tissues, an opening soon appears in the skin, where, first, a vesicle forms, from the cuticle becoming raised up from the rete malpighii. The panaritium subcutaneum has already found its way through by the fifth day, and by the twelfth the wound is generally healed. There are also cases of panaritium in which no pus is formed, but in which complete spontaneous gangrene of the tissues takes place. How is this gangrene to be explained? In inflammation, gangrene may arise through two causes: 1. The inflammatory process may from the first be so intense that the structure immediately dies; and 2. Clotting of the blood may take place and gangrene, in consequence of the arrest of the circulation. This last possibility we cannot accept in the case of panaritium, for how could such a disturbance of circulation as leads to gangrene take place so suddenly? I have already mentioned to you that the circulation in the fingers is so active that a finger may be completely cut off, except a thin bridge of skin, and the separated part may grow on again, the circulation being carried on through the bridge of skin only.

I think gangrene would take place here from the first cause. The acute inflammation is a chemical process in the tissues which is associated with dilation of the vessels with consequent exudation of serum, with increase of number of cells in the connective and fatty tissues, and with extravasation of white blood corpuscles, which come to light as pus. The most essential part of the affair, however, is the chemical process which changes, in a manner not yet understood, the inter-cellular substance and the vascular tissues. There are naturally different degrees, and these degrees are manifested in such a way that the inflammatory change and the disturbance of nutrition are small in one case, whilst in another they are extreme—indeed, the peculiar disturbance of nutrition may be so intense that the vitality of the tissues ceases. This cessation of vitality is principally of importance as regards the vascular tissues, for when once the walls of a vessel are dead—as held by Brucke—clotting of the blood readily takes place At a temperature of 1 C. (33.9 F.) no freezing takes place, and the blood, as far as it alone is concerned, may still circulate, but at this temperature the tissues of the vessels die, and clotting then takes place in consequence of their death. The like is the case in various other processes—the first in order is the death of the tissues, and the stoppage of the circulation is secondary. When gangrene takes place through the action of liquid caustics alkalies, we must look upon this in the same way as being the result of the destruction of the tissues. The conditions are similar in the case of certain animal poisons, particularly the poison of serpents, for we cannot suppose that such a disturbance of circulation is set up that gangrene follows by the mechanical injury from the teeth; it is probable that the poison itself acts chemically in such a way that it kills the tissue, and that, as a consequence of the death of these, stagnation of the blood supervenes.

